# Pathogenic Presentation of a Variant of Uncertain Significance in a Puerto Rican Patient With Wiedemann-Steiner Syndrome

**DOI:** 10.7759/cureus.37330

**Published:** 2023-04-09

**Authors:** Fabiola A Benítez Ríos, Laura F Rodríguez-Fernández, Norma J Arciniegas, Alberto Santiago Cornier, Simón Carlo

**Affiliations:** 1 Biochemistry, Ponce Health Sciences University, Ponce, PRI; 2 Pediatrics, Ponce Health Sciences University, Ponce, PRI; 3 Pediatrics, Mayaguez Medical Center, Mayaguez, PRI; 4 Public Health, Ponce Health Sciences University, Ponce, PRI; 5 Genetics, San Jorge Children's & Woman's Hospital, San Juan, PRI; 6 Genetics/Pediatrics, Mayaguez Medical Center, Mayaguez, PRI

**Keywords:** developmental and behavioural pediatrics, pediatric case report, pediatric genetics, rare genetic diseases, genetic syndromes

## Abstract

Wiedemann-Steiner syndrome (WDSTS) is an autosomal dominant disorder that is caused by mutations in the *KMT2A* gene. This case reports a two-year-old male’s diagnosis of WDSTS via a heterozygous variant of uncertain significance (VUS) (c.11735G>A(p.Cys3912Tyr). The patient's phenotypic presentation was remarkable for hypertrichosis, intellectual disability, intermittent aggressive behavior, developmental delay, failure to thrive, low weight, and the distinct facial features of long eyelashes, telecanthus, corrected strabismus, down-slanting palpebral fissures, and a wide nasal bridge with a broad tip. The importance of this case report stands on the principle of genetic evaluation in patients with ambiguous clinical presentations. In the future, molecular analysis of VUS with pathogenic clinical features can lead to targeted medical management and counseling.

## Introduction

Wiedemann-Steiner syndrome (WDSTS) (OMIM #605130) is an autosomal dominant disorder caused by *KMT2A* gene mutations [1-3]. Wiedemann first reported WDSTS in 1989 [1]. WDSTS is extremely uncommon, affecting less than one in 1,000,000 persons [1]. WDSTS is thought to affect fewer than 1,000 individuals in the United States [4] and is distinguished by developmental delay or intellectual disability, failure to thrive, feeding difficulties, prenatal and postnatal growth restriction, hypertrichosis cubiti, short stature, vertebral anomalies, and distinct facial features [5]. This syndrome’s recognizable facial features include long eyelashes, thick eyebrows, down-slanting palpebral fissures, telecanthus, strabismus, a wide nasal bridge with a broad tip, a thin vermilion of the upper lip, and a high arched palate [1,5]. Patients can also present with epilepsy, congenital heart defects, hypotonia, immune dysfunction, ophthalmologic, dental, hand, renal and uterine anomalies, and brain malformations [5]. A definitive diagnosis of WDSTS should be confirmed with genetic testing after it's suspected based on the presenting symptoms and physical exam [4]. Most patients diagnosed with WDSTS whose parents have undergone molecular genetic testing have the disorder as the result of a de novo pathogenic variant in *KMT2A* [3]. Under this circumstance, WDSTS can be inherited in an autosomal dominant manner [3]. Management of WDSTS warrants an all-encompassing evaluation, treatment, and surveillance protocol. Recommended evaluations following initial diagnosis include the measurement of growth parameters, assessments including, neurologic, developmental, neuropsychiatric if older than 12 months, musculoskeletal, including orthopedics, physical medicine, physical therapy, and occupational therapy, gastroenterology, ophthalmology, dentistry, cardiology, genitourinary, dermatologic for hypertrichosis, endocrinology, immunologic, possible sleep study, family support and resources, and continuous genetic counseling [3]. Treatment is based on patient manifestations, therefore, frequent medical follow-up for surveillance is crucial [3].

## Case presentation

**Figure 1:**
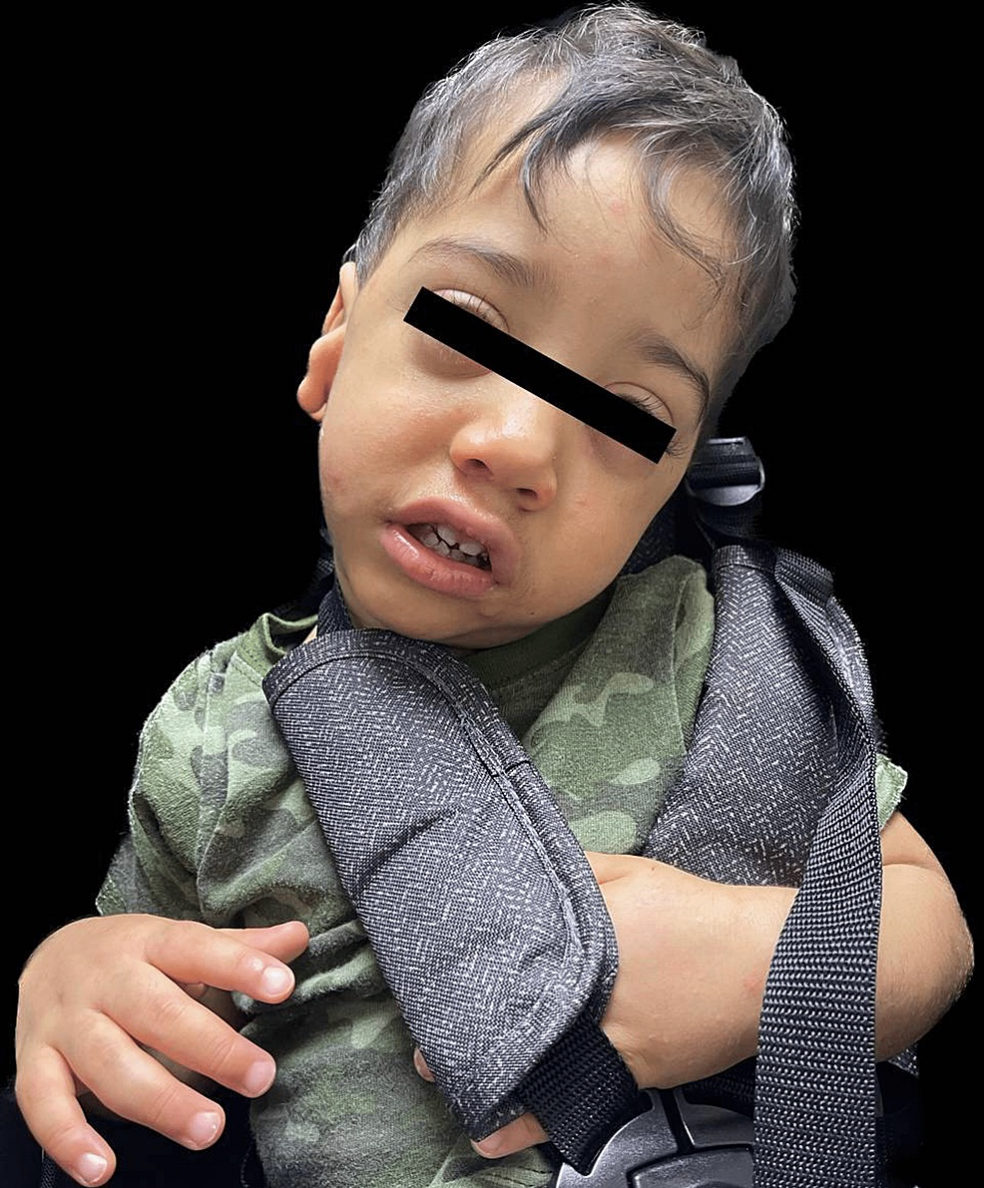
Patient presenting with Wiedemann-Steiner Syndrome facial characteristics, including long eyelashes, telecanthus, down-slanting palpebral fissures, and a wide nasal bridge with a broad tip

**Figure 2:**
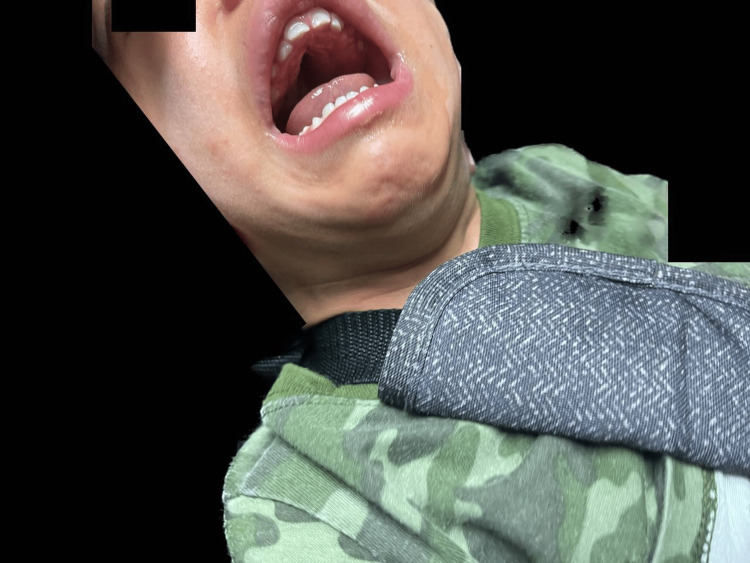
Patient presenting with a high, arched palate and dental anomalies

**Figure 3:**
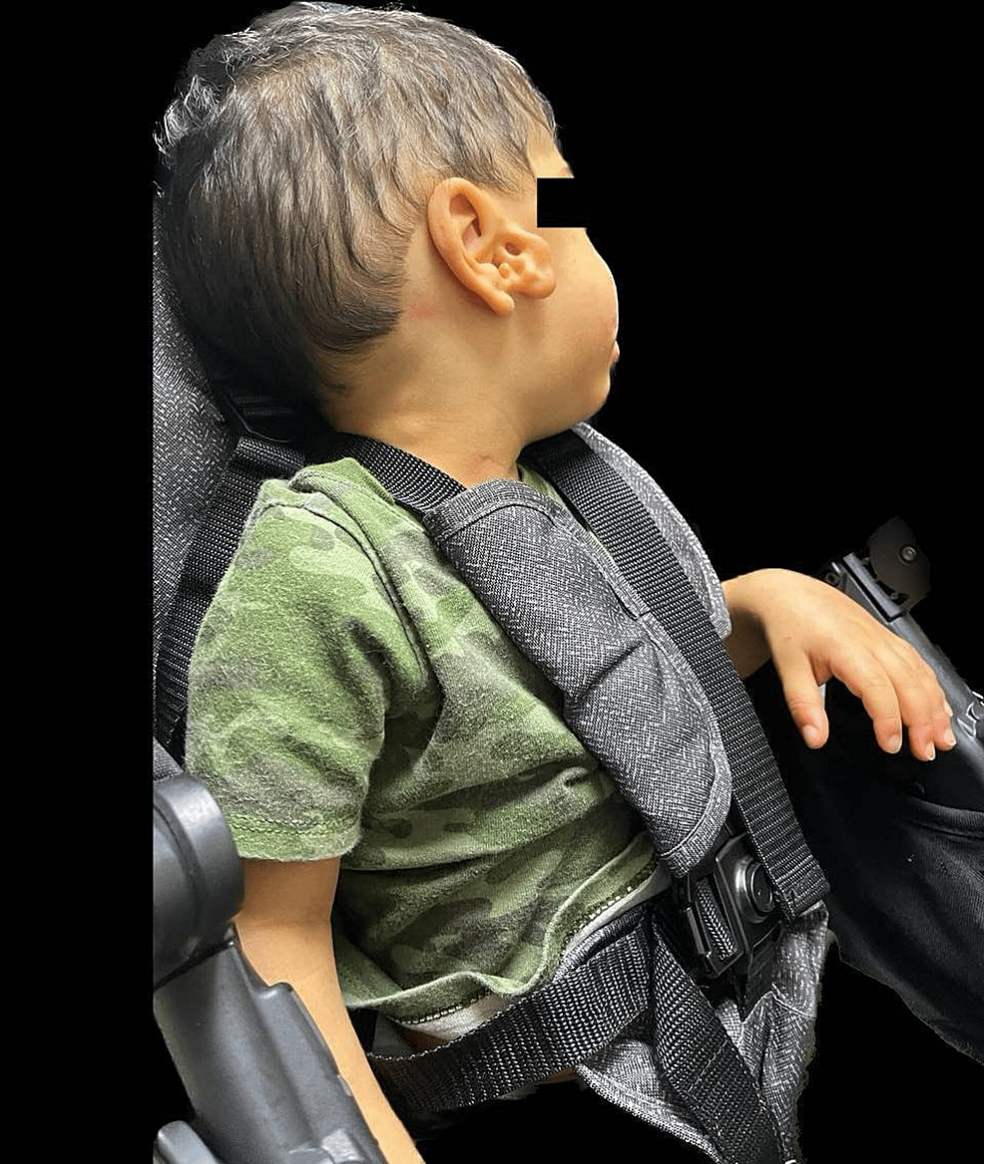
Patient’s ear malformation

We present the case of a two-year-old Hispanic male who was diagnosed with WDSTS. The patient was born vaginally to an 18-year-old Grava 2, Para 1, Abortus 0 female at 25 weeks gestation. Pregnancy was complicated by a lack of prenatal care and culminated with a precipitous delivery and premature labor. The patient had a birth weight of 780 grams, a length of 32 cm, and an occipitofrontal circumference of 20 cm. Apgar scores were five and seven at one minute and five minutes, respectively. At birth, he was intubated and transferred to the Neonatal Intensive Care Unit (NICU) due to clinical evidence of moderate respiratory distress. He remained on a ventilator for approximately two months due to oxygen dependence for appropriate clinical conditions, including adequate oxygen saturation and acid-base status. He was progressively weaned to a nasal cannula and eventually to room air due to notable improvement. Fourteen days post-birth, the patient developed a new onset of seizures. Due to this development, a urine drug screen, lumbar puncture, electroencephalogram, and metabolic workup (ammonia, lactic acid levels, and urine organic acids) were ordered. All of the results were within normal limits for age and inconclusive for any disease. Initially, the patient had a head ultrasound (HUS) performed due to prematurity and risk for intraventricular hemorrhage (IVH); initial results were normal. After a new onset of seizures, the patient had another HUS done, which confirmed a left IVH grade one. During the workup for seizures, physical medicine and rehabilitation (PMR) was also consulted due to hypotonia. The patient had a normal metabolic workup, adequate perfusion, and was under antibiotic treatment for a urinary tract infection, thus the etiology of the metabolic acidosis was suspected to be renal. He also had a cardiologic evaluation due to a history of pulmonary edema. The evaluation resulted in the discovery of a patent foramen ovale that was treated with diuretics. In terms of nutrition, he was placed on nothing by mouth (NPO) and parenteral nutrition support due to a lack of maternal breast milk. On day three, he was started on trophic feedings of formula to monitor for tolerance. The patient had to undergo several adjustments in his feedings, fluids, and medications to correct for hypoglycemia, hypernatremia, and hypokalemia. During his feeding, the patient demonstrated immature nippling skills. When he was discharged from the NICU at four months old, he was tolerating full feeds well. Over time, his foster parents stated that he was diagnosed with dysphagia and only ate mashed and puréed food. Family history was limited due to the patient’s adoption; it was only remarkable for the biological father's diagnosis of newborn onset seizures and a brother with a history of hyperactivity. Due to the patient's neonatal and developmental profile, genetic consultation and subsequent genetic tests were obtained. Exome sequencing was performed, including copy number variant (CNV) analysis and mitochondrial genome sequencing. This testing identified a heterozygous VUS in the *KMT2A* gene (OMIM*159555). The variant is a c.11735G.A (p.Cys3912Tyr) (NM_001197104.2) (gnomAD no frequency). Previously identified pathogenic variants in this gene have demonstrated the development of WDSTS [3,5]. As of now, the patient is two years old and his phenotypic expression consists of hypertrichosis, intellectual disability, developmental delay, failure to thrive, and he is underweight (corrected for gestational age (GA) weight: below the fifth percentile, corrected for GA height: 10th percentile, corrected for GA body mass index: 14.2 kg/m^2^, below the fifth percentile). In terms of behavior, the patient is easily frustrated and becomes aggressive on occasion. He also presents with the distinct facial features of long eyelashes, telecanthus, corrected strabismus, down-slanting palpebral fissures, and a wide nasal bridge with a broad tip (Figure 1). Additionally, other features that the patient presents are hypotonia, a high, arched palate with dental anomalies (Figure 2), epilepsy, immune system dysfunction (immunoglobulin A (IgA) deficiency), congenital heart defect (patent foramen ovale), retinopathy of prematurity, and an ear malformation (Figure 3). His management consists of continuous follow-up visits with endocrinology, ophthalmology, audiology, pneumology, neurology, retina specialist, cardiology, gastroenterology, genetics, dentistry, surgery, immunology, orthodontics, nutritionist, and occupational, speech, physical and musical therapies. Treatment is based on the management of symptoms.

## Discussion

This case report highlights how patients with distinct physical and developmental anomalies should undergo genetic testing when there is suspicion of an underlying genetic disorder. In patients presenting with syndromic features, genetic testing should be part of the first-line management in order to establish a proper diagnosis. As explained in the introduction, WDSTS is characterized by developmental delay or intellectual disability, failure to thrive, feeding difficulties, prenatal and postnatal growth restriction, hypertrichosis cubiti, short stature, vertebral anomalies, and distinct facial features [5]. Overlap between these signs and symptoms with other rare disorders can lead to improper clinical diagnosis of patients. Possible differential diagnoses for WDSTS include Cornelia de Lange, Coffin-Siris, Kabuki, and Nicolaides Baraitser, as they all belong to a group of syndromes akin due to variants in genes encoding components of the transcriptome and epigenetic machinery [6]. As previously stated, the patient has a heterozygous VUS in the *KMT2A* gene (OMIM*159555). The variant is a c.11735G.A (p.Cys3912Tyr) (NM_001197104.2) (gnomAD no frequency). The *KMT2A* gene, or lysine-specific methyltransferase 2A gene (OMIM*15955), spans approximately 100 kb and contains at least 21 exons in chromosome 11q23.3 [2]. It encodes a DNA-binding protein that methylates histone H3 lys4(H3K4) and positively regulates the expression of target genes [2]. RNA sequencing of this variant can lead to a better understanding of the pathophysiology of this patient’s mutation and provides a direct phenotypic/genotypic correlation for this variant. Therefore, future implications for this study include performing RNA sequencing in order to confirm the pathogenicity of this mutation, which is currently classified as a VUS. The establishment of pathogenicity for VUS helps provide a more interdisciplinary approach to patient care and aids in the vigilance of other possible conditions that the patient may be at increased risk for. An additional aim of this case report is to seed curiosity in physicians that encounter patients with distinct facial features and developmental delays. That curiosity should serve as an encouragement to seek genetic testing and reveal the underlying cause of the patient’s presentation. With improving accessibility to genetic tests, it's important to expand our diagnostic tools and improve management strategies. Limitations of this case report include the generalizability of the patient’s presentation due to his mutation being currently classified as a VUS without established pathogenicity. It would be inappropriate to state that all patients with this variant will have the same presentation and clinical implications as described in this patient. The importance of this case report renders on cementing knowledge of the understudied Puerto Rican genetic profile and this rare disease.

## Conclusions

Alterations in transcriptional and epigenetic pathways can result in syndromes with comparable dysmorphisms and phenotypic characteristics. We expect that this case report will draw attention to the use of genetic testing in clinical evaluation. Furthermore, we hope to increase awareness about the reclassification of VUS as pathogenic when it manifests phenotypically. We also intend to increase consciousness about the prevalence of rare diseases such as WDSTS, the importance of variants of uncertain significance, and the emergence of such diseases in Puerto Ricans.

## References

[1] Hirst L, Evans R (2021). Wiedemann-Steiner syndrome: a case report. Clin Case Rep.

[2] (2023). Online Mendelian Inheritance in Man, OMIM®. https://www.omim.org.

[3] Sheppard SE, Campbell IM, Harr MH (2021). Expanding the genotypic and phenotypic spectrum in a diverse cohort of 104 individuals with Wiedemann-Steiner syndrome. Am J Med Genet A.

[4] (2023). Wiedemann-Steiner syndrome. https://rarediseases.info.nih.gov/diseases/5565/wiedemann-steiner-syndrome.

[5] Shepard SE, Quintero-Rivera F (2022). Wiedemann-Steiner Syndrome. GeneReviews® [Internet].

[6] Demir S, Gürkan H, Öz V, Yalçıntepe S, Atlı EI, Atlı E (2021). Wiedemann-Steiner syndrome as a differential diagnosis of Cornelia de Lange syndrome using targeted next-generation sequencing: a case report. Mol Syndromol.

